# Investigating the psychometric properties and confirmatory factor structure of the opinions about the gifted and their education as a teacher’s attitude scale

**DOI:** 10.3389/fpsyg.2025.1568115

**Published:** 2025-10-27

**Authors:** Ainaz Shateri, Mohammad Tahan, Farank Azari

**Affiliations:** ^1^Department of Psychology, Florida International University, Miami, FL, United States; ^2^Department of Psychology and Education of Exceptional Children, University of Tehran, Tehran, Iran; ^3^Department of Psychology, Qaenat Branch, Islamic Azad University, Qaenat, Iran

**Keywords:** validity and reliability, education, teachers’ attitudes, giftedness, OGE

## Abstract

The present study aimed to determine the psychometric properties (PPs) and confirmatory factor structure of the Opinions about the Gifted and their Education (OGE) as a scale for measuring teacher’s attitudes via confirmatory factor analysis (CFA). The statistical population referenced in this research included elementary school teachers (*n* = 152), who were recruited from among the entire set of teachers working in the city of Ghaen, South Khorasan, Iran, during the 2023–2024 academic year; a multistage random sampling approach was employed. To assess the model’s adequacy, the chi-square (*χ*^2^) fit index, the *χ*^2^ to degree of freedom (df) ratio (*χ*^2^/df), the comparative fit index (CFI), the standardized root-mean-square residual (SRMR), and the relative fit index (RFI) were utilized. To establish the construct validity of the OGE scale, exploratory factor analysis (EFA) and CFA were then performed; these analyses revealed 10 factors that explained 60.12% of the common variance. Moreover, the results of the CFA demonstrated that the model exhibited an acceptable fit to the data. According to the Cronbach’s alpha coefficients, the given scale also exhibited good reliability.

## Introduction

As invaluable potential human resources in all societies, children are likely to develop into active individuals provided that their abilities and talents flourish (Agassi, 2019). Devoting much more attention to the personal differences and needs among all students in order to adapt them to their talents and abilities is thus now among the accepted principles of education (Abedi and Manani, 2013). In this line, one of the main groups of students is the gifted. In former times, giftedness was typically comparable to having a high intelligence quotient (IQ), but currently it is assumed as a multidimensional concept beyond IQ. According to some definitions, fast learning, attention control, memory efficiency, high temperament, and creativity, together with professional and educational superiority describe giftedness in a person, who is also endowed with some exceptional skills and shows intelligent behaviors (Renzulli and Reis, 2018). Gifted education is accordingly applied to some special programs and services practiced in the education of children identified with giftedness based on their abilities or talents (Afrooz and Dalir, 2017).

Like that in many other countries across the world, education in the Netherlands is progressively growing to be more inclusive (Ainscow, 2020; Ministry of Education, Culture and Science, 2016, 2021) education in the Netherlands is progressively growing to be more inclusive (Ainscow, 2020; Ministry of Education, Culture and Science, 2021) and all teachers have an important role to play in this regard (Madalińska-Michalak, 2018). In view of this, inclusive education can be a big challenge that teachers face at elementary schools.

Reflecting on the phenomenon of giftedness has drawn much attention for the past six decades. The education of exceptional talents in Iran has also been deep-rooted in history. The first centers established for gifted education at the global level have been in the fields of science and Islamic knowledge with a thousand-year history (Sattari, 2023). As a rule, there are numerous heterogeneous students in classrooms in terms of their learning abilities, knowledge levels, and skills, as well as educational needs. For example, gifted students require less repetition but more innovative teaching materials than their classmates (Little, 2018). In the Netherlands, elementary school teachers mostly perform well in educating moderate-level and poor students, while they seem to have many problems in this respect with the cognitively gifted ones (De Boer et al., 2013; Organization for Economic Co-operation and Development, 2016). Accordingly, they recurrently fail to meet the educational needs of the gifted (De Boer et al., 2013; Inspectorate of Education, 2018). Therefore, gifted students in the Netherlands are academically behind schedule as compared to their counterparts in other countries (Inspectorate of Education, 2018; Organization for Economic Co-operation and Development, 2012), which is likely to be at odds with the main purpose of education, i.e., the continuous and optimal development for each student (Ministry of Education, Culture and Science, 2021, 2025).

The teachers working in the Netherlands seem to be short of the right knowledge and attitudes toward giftedness to meet the needs of gifted students (de Boer et al., 2013; Smeets et al., 2015). Besides, they are not aware of the fact that the gifted have more educational needs. Surprisingly, they assume that gifted students are able to learn with no support (Van Gerven, 2021), which is even a common misconception in other countries (Cooper, 2009; Moon, 2009; Peterson, 2009). In addition, these teachers have no idea how to identify such students (Van Gerven, 2021). So far, many studies have been done worldwide regarding the knowledge and attitudes of teachers (particularly student teachers) toward gifted education (Cross et al., 2018; Antoun et al., 2020; McCoach and Siegle, 2007). For example, previous research on Irish (Cross et al., 2018), Finnish (Laine et al., 2019), Swedish (Allodi and Rydelius, 2008), Australian (Lassig, 2009), Lebanese (Antoun et al., 2020), and American (McCoach and Siegle, 2007; Troxclair, 2013) teachers and student teachers have accordingly demonstrated neutral but supportive attitudes toward gifted students and the special services for them among teachers. Likewise, they often had ambivalent or negative attitudes toward accelerating curricula and grouping based on students’ abilities. In this vein, investigating the levels of knowledge in German student teachers had indicated that they had many misconceptions (Heyder et al., 2018). As well, a cross-country comparative study (Matheis et al., 2017) had reported numerous false beliefs among German and Australian teachers. They had further listed uncertain attitudes raised by teachers toward gifted students in these countries. In view of that, there could be different levels of knowledge and attitudes toward gifted students and gifted education within and between countries. As a result, misconceptions and ambivalent or negative attitudes could be problematic because they were the underpinnings of educational practices (Kunter et al., 2013; Little, 2018), and could then influence some factors, such as the academic achievement and the social and emotional development of students (Miller, 2009; VanTassel-Baska and Stambaugh, 2005; Zundans, 2006).

Of note, the teachers working in the Netherlands seem to be concerned about their insufficient knowledge and unsure attitudes toward giftedness. In this line, a significant proportion of teachers had brought up a strong need for professional development for teaching gifted students (Smeets et al., 2015). Even though addressing teachers’ knowledge, attitudes, and other needs is of utmost importance during professional development activities (Desimone and Garet, 2015; Little and Housand, 2011; Guskey and Yoon, 2009), this may justify why teachers’ knowledge about gifted students is still half-finished. No professional development that agrees with teachers’ needs is thus a challenge facing teachers outside the Netherlands to tackle (Organization for Economic Co-operation and Development, 2009; 2018.

Moreover, most schools have not established clear policies or criteria regarding gifted students and some changes in educational contexts. The leading role in meeting the needs of gifted students is thus assumed by enthusiastic and committed teachers (De Boer et al., 2013; Doolaard and Oudbier, 2010). To date, little attention has been dedicated to educational knowledge and skills for gifted education in teacher training programs, as they have not been mandatory (Van Gerven, 2021). Therefore, most teachers feel like they are deficient in the required knowledge, skills, and understanding to effectively educate gifted students (de Boer et al., 2013; Van Gerven, 2021). Notably, knowledge about gifted education is typically acquired through in-service training, and teacher training centers have trivial roles in continuing professional development for gifted education (Van Gerven, 2021).

Inadequate knowledge and attitudes among teachers is thus a big problem for gifted students and the society. Teachers should accordingly understand such students and support them to fully develop their potential abilities and talents (De Boer et al., 2013). If the educational needs of gifted students are not met, their well-being is then affected and many social and emotional challenges arise (Mathijssen et al., 2018), academic achievement is lower than that expected (White et al., 2018), or some even drop out of schools (Hansen and Toso, 2007). Failure to expand the cognitive potentials of such students additionally imposes huge costs on the society in terms of productivity by the knowledge population and the gross domestic product (Minne et al., 2007; Organization for Economic Co-operation and Development, 2010). In this regard, professional development programs that help broaden teachers’ knowledge and attitudes toward gifted students and their educational needs are essential (Desimone and Garet, 2015; Little and Housand, 2011), as they simultaneously meet teachers’ needs and enhance gifted education (Thurlings and Den Brok, 2017). Against this background, the present study was to determine the psychometric properties (PPs) of the Opinions about the Gifted and their Education (OGE) as a teacher’s attitude scale.

## Methods

The statistical population in this study encompassed the elementary school teachers working in the city of Ghaen, South Khorasan, Iran, in the 2023–2024 academic year, selected by multistage random sampling. Out of 260 questionnaires distributed among the participants, 152 cases were finally analyzed in the course of the hypothesis-testing of the research tool. Sampling in this study was also organized into two phases:

1 Research tool translation and adjustment

As first, the OGE scale (Gagné and Nadeau, 1991) was translated and revised using back-translation method (Brislin, 1970), and then it was submitted and then it was submitted to a panel of experts to comment on the appropriateness of its items for the Iranian society as well as its applicability. Conclusively, no item was found to conflict with the Iranian society, the experts advocated this scale, and some faculty members also confirmed its content validity and sociocultural compatibility.

2 Validity and reliability

To assess the validity and reliability of the OGE scale, 152 teachers were selected out of those working in the city of Ghaen, South Khorasan, Iran, by multistage random sampling. Then, the given scale was provided to them to fill in. In order to meet the study objectives, viz., investigating the PPs of the scale, the content validity (expert opinions), construct validity (confirmatory factor analysis: CFA), and internal consistency (Cronbach’s alpha coefficient) were considered. The SPSS Statistics (ver. 28) and AMOS (ver. 26) software packages were also utilized for data analysis purposes. To report the CFA outcomes, some indices, including the Chi-squared (*χ*^2^) fit index, the normed *χ*^2^ measure or the *χ*^2^ to the degree of freedom (df) ratio (*χ*^2^/df), the comparative fit index (CFI), the incremental fit index (IFI), the goodness-of-fit index (GFI), and the root-mean-square error of approximation (RMSEA) were employed. Of note, *χ*^2^ was taken into account as a measure of the overall fit of the model with the data.

### Research tool

In this study, the OGE scale was administered as a valid and reliable data collection tool to elicit teachers’ attitudes toward giftedness. It contained 34 items within six components, namely, acceleration, needs, support, elitism, value, and opposition, scored based on a five-point Likert-type scale (viz., totally disagree, sometimes disagree, neutral, sometimes agree, totally agree). The Cronbach’s alpha coefficient for each component was 0.70–0.83. The OGE scale was further developed in Google Forms and shared with teachers on the social media platforms.

## Results

According to Table 1, the mean±SD (the total score) of the OGE scale was 3.33 ± 0.40. As well, this value for the sub-scale of acceleration was 2.99 ± 0.79. The mean±SD for the sub-scales of needs, support, elitism, value, and opposition were also equal to 2.71 ± 0.62, 4.07 ± 0.76, 3.45 ± 0.56, 3.50 ± 0.66, and 3.59 ± 0.46, respectively (Table 2).

**Table 1:** Mean, standard deviation (SD), minimum, and maximum scores of the OGE scale and its sub-scales.

	Components	Mean	SD	Maximum	Minimum
Sub-scales	Acceleration	2.9912	0.79637	5	1
	Needs	2.7146	0.62895	4.75	1.25
	Support	4.0789	0.76795	5	1.67
	Elitism	3.4521	0.56296	5	2.14
	Value	3.5039	0.66431	5	1.80
	Opposition	3.5995	0.44604	5	2.13
	Total score	3.3355	0.40821	4.94	2.09

**Table 2:** Kaiser-Meyer-Olkin (KMO) test and Bartlett’s test coefficients.

Groups	KMO test value	Bartlett’s test value	Significance level
Factors	0.736	1493.307	0.001

Considering the KMO value, which was greater than 0.6, the OGE items suited for factor analysis.

As presented in Table 3, factor analysis using principal component analysis (PCA) led to the emergence of 10 factors with the eigenvalue above 1, and such factors further explained 60.120% of the common variance. Table 3 also outlines the factors extracted from the OGE scale, using varimax rotation in factor analysis and the principal components.

**Table 3:** Characteristics of 10 factors extracted of from the OGE scale.

Factors	Eigenvalues	Explained variance percentage	Cumulative variance percentage
1	5.429	15.967	15.967
2	3.920	11.529	27.496
3	2.147	6.314	33.810
4	1.584	4.657	38.468
5	1.399	4.115	42.582
6	1.307	3.844	46.427
7	1.275	3.750	50.176
8	1.160	3.411	53.587
9	1.141	3.357	56.944
10	1.080	3.175	60.120

In accordance with Table 4, the exploratory factor analysis (EFA) resulted in the formation of 10 factors that accounted for 60.12% of the common variance and all the OGE scale items with favorable factor loadings from 0.3 to 0.8. These findings correspondingly proved the construct validity of the translated version of this scale for the Iranian society.

**Table 4:** Factors extracted from the OGE scale using varimax rotation in factor analysis.

Item/Factor	1	2	3	4	5	6	7	8	9	10
1	0.566									
2	0.564									
3	0.713									
4	0.665									
5	0.234									
6	0.458									
7		0.617								
8		0.365								
9		0.607								
10		0.739								
11		0.486								
12			0.602							
13			0.463							
14			0.643							
15			0.307							
16			0.569							
17				0.583						
18				0.518						
19				0.761						
20				0.333						
21					0.331					
22					0.561					
23					0.639					
24					0.510					
25						0.515				
26						0.470				
27						0.769				
28						0.395				
29							0.709			
30							0.497			
31								0.443		
32								0.783		
33									0.824	
34										0.795

**Figure 1:**
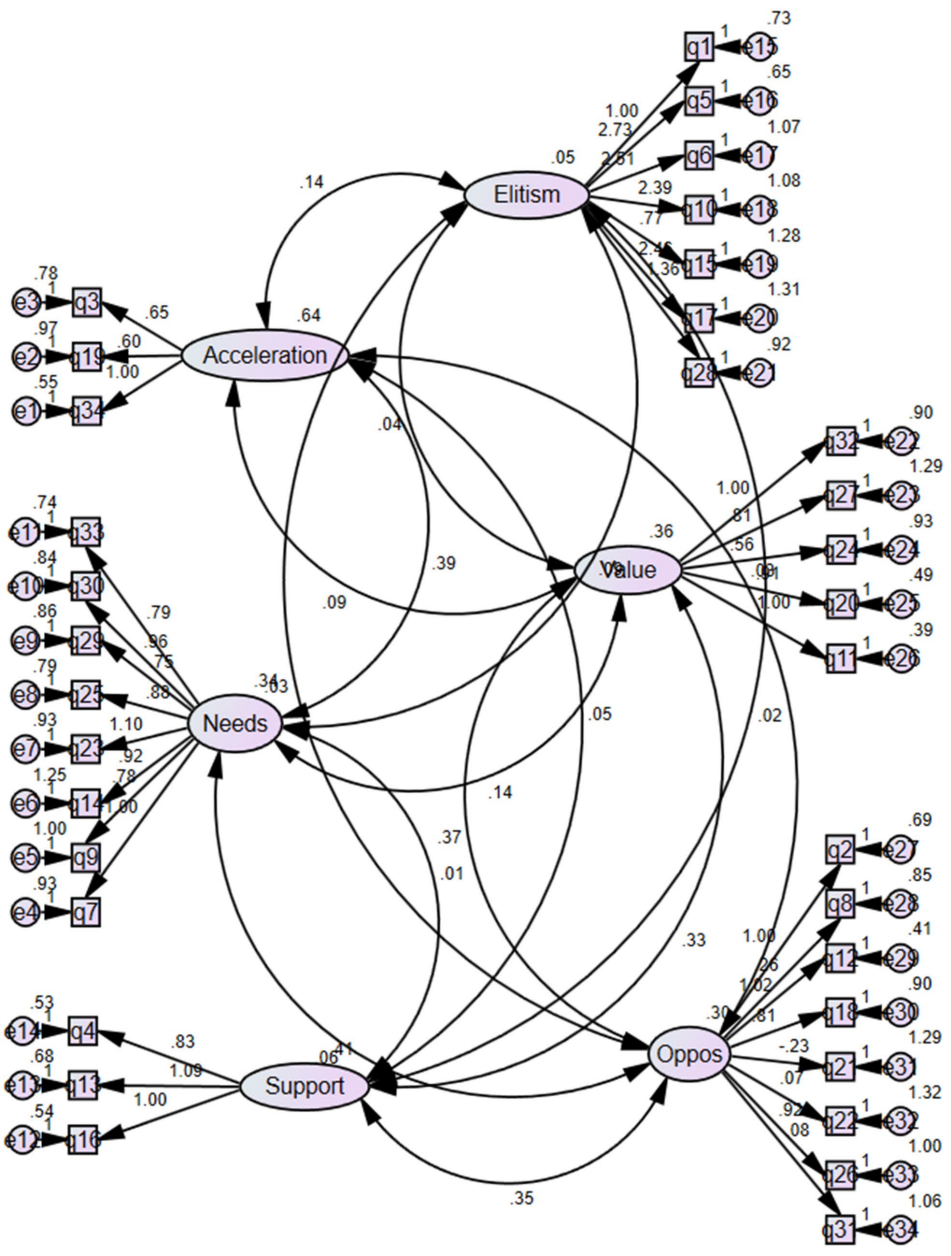
Factor structure of the OGE.

As illustrated in Figure 1, the factor loadings of all the scale items were over 0.30 in the CFA, which seemed to be appropriate, and the related factor also had a significant positive loading at the *p* < 0.001 level. Moreover, the highest and lowest factor loadings were associated with Item no. 27 (1.29) and Item no.11 (0.39), respectively.

According to Table 5, the CFA results for the mentioned scale revealed the values of the GFIs, including *χ*^2^ = 844 with the df = 512, the normed *χ*^2^ measure (*χ*^2^/df) = 1.64, the GFI = 0.765, the adjusted GFI (AGFI) = 0.727, the IFI = 0.701, the CFI = 0.688, and the RMSEA = 0.066. Accordingly, the OGE scale was consistent with the proposed criteria of the appropriate values of the fit indices (Table 6).

**Table 5:** CFA fit indices.

Fit indices	*χ* ^2^	df	χ^2^/df	GFI	AGFI	IFI	CFI	RMSEA
OGE	844.04	512	1.64	0.765	0.727	0.701	0.688	0.066

**Table 6:** Internal correlation coefficients of the OGE scale components with total score.

Components	Correlation coefficient with total score	Significance level
Acceleration	0.615	0.000
Needs	0.716	0.000
Support	0.497	0.000
Elitism	0.718	0.000
Value	0.662	0.000
Opposition	0.738	0.000

In this respect, the internal correlation of the scores of the components with the total score of the OGE scale was found to be favorable and fitting (*p* < 0.01).

As presented in Table 7, the Cronbach’s alpha coefficient for the whole OGE scale (0.83) and its sub-scales was between 0.70 and 0.83, which implied their acceptable reliability.

**Table 7:** Reliability coefficients of the OGE scale.

OGE scale	Cronbach’s alpha coefficient
Acceleration	0.79
Needs	0.77
Support	0.83
Elitism	0.74
Value	0.75
Opposition	0.70
Acceleration	0.83

## Discussion and conclusion

The present study was to determine the PPs of the OGE scale, as an important part of teachers’ professional competence. The study results accordingly demonstrated that most teachers were aware of the qualities of giftedness and realized that it was something beyond IQ in students. The teachers’ attitudes toward gifted students and their educational needs correspondingly provided a positive overall picture. Most of the OGE scale items had high mean scores, which denoted positive or very positive attitudes. Teachers also valued gifted students and supposed that they had the same right to a supportive learning environment as others. Even with teachers’ overall positive attitudes toward giftedness in students and their needs, they were facing some conflicting attitudes toward educational adaptations, such as accelerating curricula and grouping based on abilities and talents. They also had some common misconceptions about social maladjustment and knowledge gaps on account of accelerated curricula. Previous research on Irish (Cross et al., 2018), Finnish (Laine et al., 2019), Swedish (Allodi and Rydelius, 2008), Australian (Lassig, 2009), Lebanese (Antoun et al., 2020), and American (McCoach and Siegle, 2007; Troxclair, 2013) teachers or student teachers had harmoniously shown that they generally had positive attitudes toward gifted students and special services for them, but ambivalent or negative attitudes toward accelerating curricula and grouping with reference to their abilities. In spite of this, Hattie (2009) and Rogers (2015) had found the significant effectiveness of such strategies, viz., accelerating curricula and grouping based on abilities.

The way giftedness is thus perceived and identified largely depends on the dominant culture (Sternberg, 2007). Educational and psychological knowledge can further support teachers in the face of common misconceptions about the gifted (Heyder et al., 2018), and then help them perform better in making educational adaptations needed by gifted students (Little, 2018). Over and above knowing about the content and the way to deliver it as well as creating supportive learning environments for all students, teachers’ attitudes are a portion of their professional competence (Kunter et al., 2013). Attitudes also refer to the feelings and cognitive beliefs in a person about something or someone and the behaviors received in response (Stern and Keislar, 1975). They can accordingly shape different behaviors at the individual, interpersonal, and social levels (Bohner and Wänke, 2002). Teachers’ attitudes toward giftedness and gifted education consequently affect the performance of students because such attitudes have an effect on teachers’ behaviors in classrooms (Ajzen, 1991; Berman et al., 2012). For example, Berman et al. (2012) had found that teachers’ attitudes toward gifted students could influence their willingness to teach them as well as the adoption of the right teaching methods and other educational strategies for gifted students. In addition, teacher’ attitudes can manipulate attitudes, performance, creativity, and social and emotional development in gifted students (Miller, 2009; VanTassel-Baska and Stambaugh, 2005; Zundans, 2006).

In view of that, the OGE scale has been designed to reflect on the elementary school teachers’ attitudes toward gifted students and their educational needs. Based on the analysis of the data obtained from 152 teachers, this scale had good internal validity and reliability.

Although the confirmatory factor analysis supported the factorial structure of the adapted OGE scale, it is noted that the values for the Comparative Fit Index (CFI = 0.688) and the Incremental Fit Index (IFI = 0.701) were below the conventional threshold of 0.90. This pattern is not uncommon in validation studies with complex models and modest sample sizes (Hu and Bentler, 1999; Kenny, 2020). The relatively lower values may be attributable to several factors. First, the sample size (*n* = 152), while adequate for preliminary validation, can influence the stability of these particular indices. Second, the cultural and linguistic adaptation process, though rigorous, may introduce minor nuances in item interpretation that are reflected in the model’s fit. Finally, the original multi-dimensional structure of the OGE scale is inherently complex. It is important to emphasize that other absolute and parsimony-adjusted fit indices, such as the *χ*^2^/df ratio (1.64) and the RMSEA (0.066), were within acceptable ranges, indicating a good fit between the model and the observed data (Schermelleh-Engel et al., 2003). Furthermore, the strong reliability coefficients and the clear factor structure derived from EFA provide robust evidence for the validity and utility of the adapted scale, ensuring that the conclusions drawn are well-founded.

Among the limitations in this study was that the teachers were aware of the scale subject and the research context, so they did not possibly express their attitudes with honesty. The design and combination of professional development activities for teachers to meet their knowledge, attitudes, and professional development needs can be thus addressed in future research to ultimately facilitate the better education of gifted students. Even with the existing limitations, the present study investigated elementary school teachers’ attitudes toward the gifted students and their educational needs in Iran.

## Data Availability

The raw data supporting the conclusions of this article will be made available by the authors, without undue reservation.
